# Dynamics of enhancers in myeloid antigen presenting cells upon LPS stimulation

**DOI:** 10.1186/1471-2164-15-S10-S4

**Published:** 2014-12-12

**Authors:** Alexis Vandenbon, Shunsuke Teraguchi, Osamu Takeuchi, Yutaka Suzuki, Daron M Standley

**Affiliations:** 1 Laboratory of Systems Immunology, Immunology Frontier Research Center, Osaka University, Suita, 565-0871, Japan; 2 Immuno-Genomics Research Unit, Immunology Frontier Research Center, Osaka University, Suita, 565-0871, Japan; 3 Quantitative Immunology Research Unit, Immunology Frontier Research Center, Osaka University, Suita, 565-0871, Japan; 4 Laboratory of Infection and Prevention, Institute for Virus Research, Kyoto University, Kyoto, 606-8507, Japan; 5 Department of Medical Genome Sciences, Graduate School of Frontier Sciences, The University of Tokyo, Kashiwa, 277-8568, Japan

## Abstract

**Background:**

Recent studies have underscored the role of enhancers in defining cell type-specific transcriptomes. Cell type-specific enhancers are bound by combinations of shared and cell type-specific transcription factors (TFs). However, little is known about combinatorial binding of TFs to enhancers, dynamics of TF binding following stimulation, or the downstream effects on gene expression. Here, we address these questions in two types of myeloid antigen presenting cells (APCs), macrophages and dendritic cells (DCs), before and after stimulation with lipopolysaccharide (LPS), a potent stimulator of the innate immune response.

**Results:**

We classified enhancers according to the combination of TFs binding them. There were significant correlations between the sets of TFs bound to enhancers prior to stimulation and expression changes of nearby genes after stimulation. Importantly, a set of enhancers pre-bound by PU.1, C/EBPβ, ATF3, IRF4, and JunB was strongly associated with induced genes and binding by stimulus-activated regulators. Our classification suggests that transient loss of ATF3 binding to a subset of these enhancers is important for regulation of early-induced genes. Changes in TF-enhancer binding after stimulation were correlated with binding by additional activated TFs and with the presence of proximally located enhancers.

**Conclusions:**

The results presented in this study reveal the complexity and dynamics of TF- enhancer binding before and after stimulation in myeloid APCs.

## Background

The control of gene expression plays a central role in nearly all biological processes. Transcription initiation is regulated on a number of levels, including modification of epigenetic markers and recruitment of RNA polymerase by transcription factors (TFs) [1]. Enhancers can be functionally defined as short genomic regions which regulate expression of genes, often over long distances. It is well established that enhancers play a key role in the regulation of gene expression [2,3]. Recent developments in sequencing techniques have enabled high-resolution investigation of a wide variety of histone modifications, and their functional annotation [4,5]. Enhancers have been shown to be marked by high amounts of the histone modification H3K4me1 [5,6], and recent estimates suggest that several hundred thousand enhancers exist in the human and mouse genomes [6,7].

However, despite the identification of master regulators in several cell types, and technical advances in molecular biology, much remains obscure. For example, the degree to which cell type-specific enhancers are dependent solely on pioneer factors or master regulators is poorly understood. Specific combinations of TFs that bind to enhancers might play key roles in regulating genes involved in biological processes, but which TF combinations control which processes is generally unknown. Finally, the dynamics in the binding of regulatory elements following stimulation, as well as the interactions between these elements, have not been well described.

Here, we address these issues using myeloid APCs (macrophages and DCs). These cells represent a first line of defence against pathogens as part of the innate immune system, and play a role in the subsequent activation of the adaptive immune system. A number of recent studies have emphasized a central role of the lineage-determining Ets family member PU.1 in defining cell type-specific enhancers in APCs. Binding of PU.1, in combination with a small set of cell type-restricted, lineage-determining factors, is necessary for defining macrophage-specific H3K4me1-marked regions during differentiation, and the binding of PU.1 in macrophages co-occurs with the binding of stress-inducible TFs, such as NF-κB and IRFs [8,9]. It has also been shown that in terminally differentiated macrophages so-called latent enhancers become bound by stimulus-activated and lineage-determining TFs only after stimulation [10]. A similar central role of PU.1 as a master regulator defining cell type-specific enhancers and regulating the response to immune stimuli has been shown in DCs [11].

The myeloid APCs analysed in this study present a useful system for integrative analysis since there is an abundance of genome-wide data available for these cells. Here, we generated RNA-seq data as a measure of gene expression and transcription start site sequencing (TSS-seq) data [12] as a measure of transcription initiation events, and analysed it in combination with publicly available ChIP-seq data for various histone modifications [8,13], 24 TFs and RNA polymerase II (Pol2) [11]. We used these data sets to define enhancers on a genome-wide level, and to carry out a detailed analysis of enhancer-TF interactions. We found that regions with enhancer-like features were bound by a variety of sets of principal TFs. Specifically, we found that one class of enhancers was bound even before stimulation by PU.1, C/EBPβ, ATF3, IRF4, and JunB (here referred to as "class H_1 _enhancers"). This class was strongly associated with genes that have induced expression following immune stimulation with LPS. After stimulation, the same enhancers were then preferentially bound by activated TFs, such as NF-κB, IRFs, and STAT family TFs. This suggests that the behaviour of genes after stimulation is, to some degree, already decided by the TFs binding to nearby enhancers before stimulation. On the other hand, we also found a considerable degree of change in TF binding to enhancers after stimulation. One change, the transient loss after LPS stimulation of ATF3 binding at H_1 _enhancers, appears to control a set of early induced genes. Our results suggest that such changes might be governed by interactions between activated TFs and principal binding TFs, as well as between proximally located enhancer pairs.

## Methods

**Figure 1:**
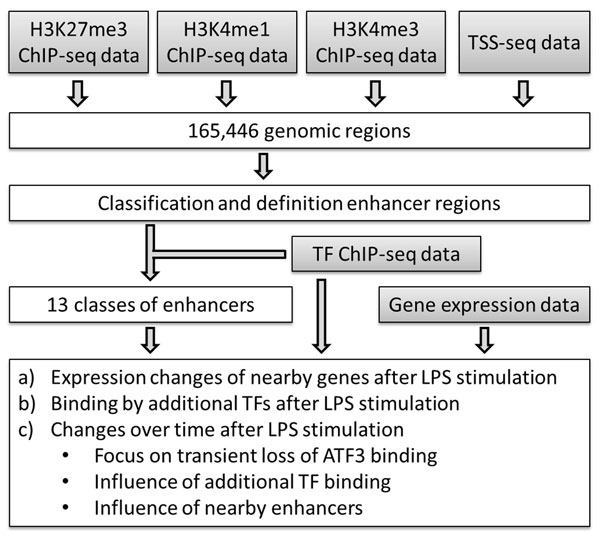
**Flowchart of the main steps in this study**. Step 1 is the identification of genomic regions marked by one or more histone modifications or transcription initiation events. In step 2 genomic regions are clustered according to their features and enhancer regions are defined. In step 3, enhancers are further classified into 13 classes according to the pre-stimulation binding by six principal TFs. We perform various more detailed analyses on these 13 classes of enhancers in step 4, including analysis of gene expression of nearby genes, binding by additional TFs, and changes in binding by principal TFs after stimulation. Main input data are marked in grey.

We refer to Figure 1 for a flowchart of the main steps of this study. Below is a detailed description of the steps and results.

### Acquisition and processing of high-throughput sequencing data

For TSS-seq data analysis, we prepared peritoneal exudate cells from C57BL/6 mice 3 days after injection with 2ml 4% thioglycolate medium (Sigma). Cells attached on the culture dish were stimulated with 100 ng/ml LPS from S. Minnesota Re595 (Sigma) for 4 h, followed by the extraction of total RNAs with Trizol (Life Technologies). TSS-seq was performed on these RNAs using the procedure described in [12]. RNA-seq data was taken from macrophages at three time points (0h, 1h, and 4h after stimulation with LPS). The short-read sequence archive data are registered in the DNA Data Bank of Japan (DDBJ) under accession nos. [DDBJ:DRA001207] and [DDBJ:DRA001208]. Reads were mapped to the mm9 genome using ELAND [14] and uniquely mapped reads with at most 2 mismatches to the reference genome were used for further analysis.

For ChIP-seq data, public ChIP-seq reads for H3K4me3 (0h: GSM470558, 4h: GSM470559), H3K27me3 (0h: GSM470560, 4h: GSM470561), and H3K4me1 (0h: GSM487452) taken from bone marrow-derived macrophages (BMDMs) [8,13] were downloaded from the NCBI Gene Expression Omnibus and mapped to the mm9 genome using SOAP2 [15]. ChIP-seq data for PU.1 (GSM487450), C/EBPβ (GSM537985), and Pol2 (GSM470562) was processed in the same way.

### Classification of genomic regions and definition of enhancer regions

In order to define potentially functional genomic regions from TSS-seq and ChIP-seq for H3K27me3, H3K4me1, and H3K4me3, the number of mapped tags in the mouse genome was counted in bins of 200 bps in steps of 200 bps. For the TSS-seq reads this was done in a strand-specific way. The position of mapped ChIP-seq reads was shifted by 75 bp in the 3' direction. Significant peaks were detected using a Poisson distribution-based p value, using a threshold p value of 1e-6. Regions containing significant levels of one or more features were merged if they were less than 500 bps separated. The weighted average of ppm reads was used to define the central bin of each region, and the 10 bins upstream and downstream of this central bin (total of 21 bins, 4.2kb). Regions containing mapped TSS-seq reads were oriented so that the majority of tags were located on the "+" strand. We excluded regions containing significant levels of mapped TSS-seq reads but lacking any histone modifications. Such loci were especially enriched in 3' UTR regions, and might represent reads originated by recapping, or by genuine transcription initiation [16,17]. This procedure resulted in 165,446 genomic regions associated with significant levels of one or more epigenetic or transcriptomic features.

**Figure 2:**
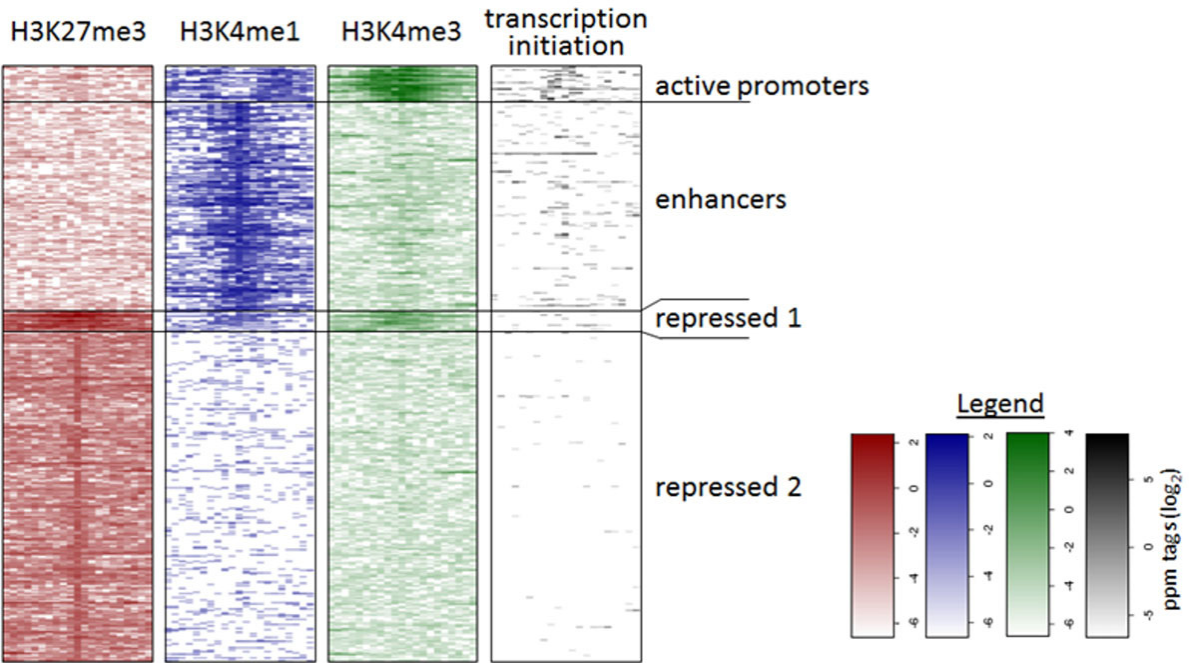
**Clustering of genomic regions and definition of enhancer regions**. Heatmap representing the clustering of genomic regions into 4 classes according to their H3K27me3, H3K4me1, H3K4me3, and transcription initiation profile.

Regions were marked as overlapping with TSS regions, 5' UTRs, 3' UTRs, exonic regions, or intronic regions, in this order of preference, if they overlapped with these features based on Refseq annotations as available in the UCSC database [18,19]. Regions not overlapping with any of these features were marked as intergenic. K- means clustering was used to identify enhancer-like genomic regions according to histone modifications and transcription initiation events (Figure 2).

### Analysis of TF binding to enhancer regions

Genome-wide binding regions of 24 TFs (Ahr, Atf3, Cebpb, Ctcf, E2f1, E2f4, Egr1, Egr2, Ets2, Hif1a, Irf1, Irf2, Irf4, Junb, Maff, Nfkb1, PU1, Rel, Rela, Relb, Runx1, Stat1, Stat2, Stat3) in bone marrow-derived dendritic cells (BMDCs) before and after stimulation with LPS [11] were obtained from the Genome Expression Omnibus (accession number GSE36104). We used ChIP-seq-based peak scores as reported in the original study [11], as an indication of TF binding throughout the genome. For each TF at each available time point, we associated peak scores with the center of the reported peak regions, and assigned them to the corresponding bins of 200 bps for all 165,446 regions defined above. Bins not including a peak region center received a score of 0.

The overall binding tendencies of TFs to enhancers was evaluated using the highest peak score assigned to the central 11 bins of 200 bps (corresponding to the region - 1.1kb to +1.1kb surrounding the region center). Table 1 shows the percentage of enhancer regions having a score higher than or equal to 26.9, the threshold score used in original study [11], for each TF at time point 0h.

**Table 1:** Principal TFs binding active promoters and enhancer regions.

Regulator	Bound enhancers (%)
PU.1	38.0
C/EBPβ	26.0
JunB	14.1
IRF4	12.1
CTCF	8.9
ATF3	8.4

The percentage of bound enhancers for a set of 6 principal TFs is shown, based on ChIP-seq data (11). TFs bound to more than 5% enhancers are shown.

### Clustering of enhancers by the binding of principal TFs

Enhancers were clustered using the binding by PU.1, C/EBPβ, CTCF, Atf3, Irf4, and Junb at time point 0h. For each of these 6 TFs, the highest score over the central 11 bins of 200 bps was assigned to each enhancer. Scores higher than 26.9 were set to 26.9. Enhancers were clustered using k-means clustering using these scores. The optimal number of clusters was estimated to be 13 using the Gap statistic method [20].

For the analysis of changes over the time points after stimulation, the same 13 clusters based on the time point 0h clustering were used to cluster enhancer regions. This was done by assigning regions to the most proximal cluster, in terms of Euclidian distance.

### Gene expression analysis

From the RNA-seq data taken from macrophages at three time points (0h, 1h, and 4h after stimulation with LPS), RPKM (reads per kilobase per million reads) values were calculated for all genes, and subjected to quantile normalization. We identified 2,188 genes with at least 3-fold differential expression over the three time points, and at least one time point with RPKM higher than 1. These genes were clustered into 4 classes ("early induction", "late induction", "gradual induction", and "repression") based on their log(RPKM) values using hierarchical clustering. In addition, we defined a class of genes with unchanged expression after stimulation as the 5,000 genes with the smallest fold changes over the three time points.

Micro-array gene expression data for ATF3 knock-out (KO) and wild-type (WT) BMDMs was obtained from ArrayExpress (ID: E-TABM-102) [21]. RMA normalized probe intensities were averaged over duplicate experiments, and gene expression levels were calculated by averaging over probes.

### Assigning genes to enhancer regions

Enhancer regions were naively assigned to the most proximal gene, based on the distance in bases between the center of the enhancer region and the gene's TSS. Multiple enhancers can be assigned to the same gene.

For the analysis of associations between enhancer classes and sets of genes with a particular expression profile (see section Gene expression analysis), we first counted for each set of genes, the number of assigned enhancer regions of each enhancer class. Next, a Z score was calculated based on this count and the average and standard deviation of expected counts obtained from 100 sets of enhancers with randomly shuffled enhancer class indices.

### Further supporting analysis and data availability

Distances between pairs of enhancers were calculated as the distance in bases between their centers. GC content and CpG scores in regions were calculated in bins of 200 bps. TFBS motif enrichment was performed as described in Additional file 1 (Supplementary Material).

The short-read sequence data obtained for this research are registered in the DDBJ under accession nos. [DDBJ:DRA001207] (RNA-seq data) and [DDBJ:DRA001208] (TSS-seq data).

## Results

### Identification of enhancer regions using epigenetic markers and transcription initiation events

Given the known chromatin signature associated with active and inactive promoters and enhancer regions, we detected 165,446 genomic regions based on statistically significant enrichment of epigenetic markers, H3K4me3, H3K27me3, and H3K4me1 (obtained from ChIP-seq data using BMDMs) along with transcription initiation events (from newly obtained TSS-seq data from thioglycollate-elicited peritoneal macrophages). To identify enhancer regions among these candidate regions, we used k-means clustering which resulted in 4 distinct clusters (Figure 2 and Materials and Methods section). The cluster ("active promoters"; 9,586 regions) with high levels of transcription initiation events also showed high levels of H3K4me3 and lower levels of H3K4me1 (Supplementary Figure S1A in Additional file 1), fitting well with the known characteristics of active promoters. The "enhancer" cluster (58,370 regions), on the other hand, lacked high levels of transcription initiation and H3K4me3, but contained high levels of H3K4me1, and thus is consistent with previous findings. There is a clear difference in the profile of the H3K4me1 marker; in the promoter group H3K4me1 is limited to regions surrounding the strong H3K4me3 peak, which is absent in the enhancer cluster (Supplementary Figure S1A in Additional file 1). Other properties of the "active promoter" and "enhancer" regions are also consistent with known characteristics (see Supplementary Material and Supplementary Figures S1B-C in Additional file 1). The two remaining clusters were marked by high levels of the repressive marker H3K27me3, either in combination with low levels of H3K4me1 and H3K4me3 ("repressed 1"; 5,394 regions) or by the repressive marker H3K27me3 alone ("repressed 2"; 92,096 regions). For the remainder of this paper we will focus on the analysis of the "enhancer" regions.

### Clustering of enhancer regions according to binding by 6 principal transcription factors

**Figure 3:**
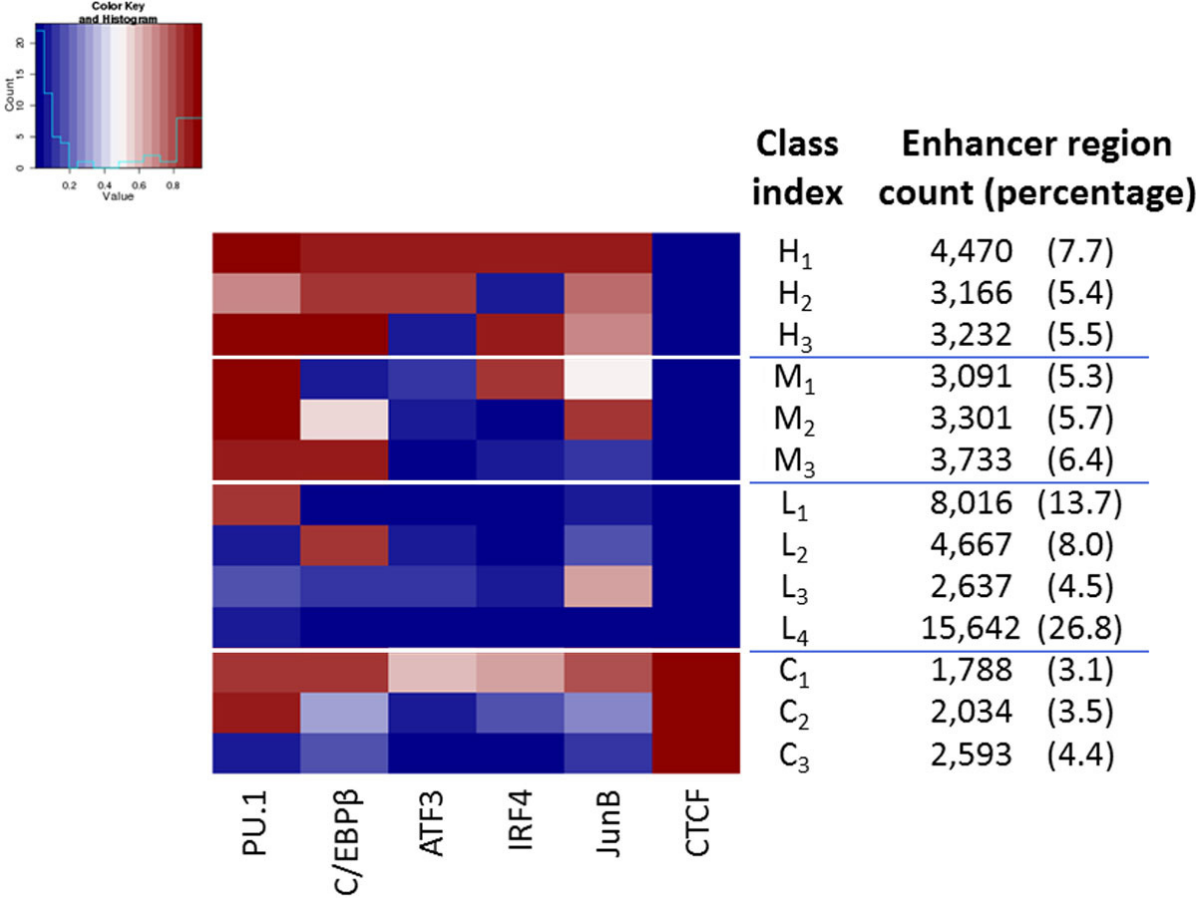
**Clustering of enhancer regions according to bound TFs**. For the 13 enhancer classes (rows) the average normalized peak score for the 6 principal TFs (columns) is shown. Colors represent the average rates of binding of each TF in each class of enhancers, with red, white, and blue colors indicating high, intermediate, and low rates of binding, respectively. Columns on the right of the heatmap show for each class the index, the number of regions in the class in unstimulated cells, and the corresponding percentage of the total set of enhancers.

We verified the binding of TFs to enhancer regions in unstimulated cells using ChIP-seq data for a set of 24 TFs that are highly expressed in BMDCs [11] (Materials and Methods section). We found that in addition to PU.1 and C/EBPβ, a number of other TFs are also associated with a substantial fraction of enhancers (Table 1 and Supplementary Table S1 in Additional file 1). In particular, Atf3, Irf4, and Junb were significantly bound to more than 5% of enhancers and promoters. Interestingly, the insulator-binding protein CTCF was associated with 8.9% of enhancers (and 22.3% of active promoters), consistent with observations made in recent studies [7,22,23]. In order to reveal the pattern of combinatorial TF binding to enhancers and analyze their distinctive properties, we further clustered enhancer regions in terms of binding by 6 principal TFs (PU.1, C/EBPβ, ATF3, IRF4, JunB, and CTCF). Again, using k- means clustering, with the optimal number of clusters based on the Gap statistic method [20] (Materials and Methods section), enhancers were clustered into 13 classes (Figure 3). We roughly divided these 13 classes of enhancers into 4 groups according to the number of principal TFs binding them. The 13 classes were specified here using a 2-character index, with the first index reflecting the number of principal TF binding them; H (Highly bound), M (Medium bound), L (Lowly bound), and C (bound by CTCF). The second index indicates a further subdivision and ranged from 1-4. This clustering allows us to make several observations. First, there exists considerable variety in the sets of TFs binding enhancer regions. Class H_1 _enhancers are bound by PU.1, C/EBPβ, ATF3, IRF4, and JunB, while class L_4 _enhancers are bound by none of the 6 principal TFs. Compared to class H_1 _enhancers, class H_2 _and H_3 _enhancers lack IRF4 and ATF3 binding, respectively. Other classes are bound by other combinations of TFs. Second, most of the enhancer classes are bound by PU.1 and C/EBPβ as a pair or in combination with other principal TFs. These enhancers fit well with the notion that macrophage- and DC-specific enhancers are defined by PU.1 as a master regulator in combination with C/EBPβ as a lineage-specific TF. However, in addition to a large subset of enhancers being bound by none of the 6 principal TFs (class L4; 15,642 regions; 26.8%), a number of classes lack binding by PU.1 (L2), or C/EBPβ (L_1 _and M_1_), or both (L_3 _and C_3_). The existence of classes L_3 _and C_3 _suggest that JunB or CTCF are able to bind H3K4me1-marked regions even in the absence of master regulator PU.1 and C/EBPβ. Classes L_1 _and L_2 _suggest that H3K4me1-marked regions can be bound by either PU.1 or C/EBPβ in the absence of any of the other principal TFs. TFBS sequence motif analysis confirmed many of the observed binding tendencies (see Supplementary Material and Supplementary Figure S2 in Additional file 1). Together, these results suggest considerable variety in TF binding at enhancer regions, and that a substantial fraction of enhancers differ in their TF binding from "typical" myeloid APC enhancers.

### H_1 _class enhancers are especially associated with induced genes and binding by TFs activated after LPS stimulation

**Figure 4:**
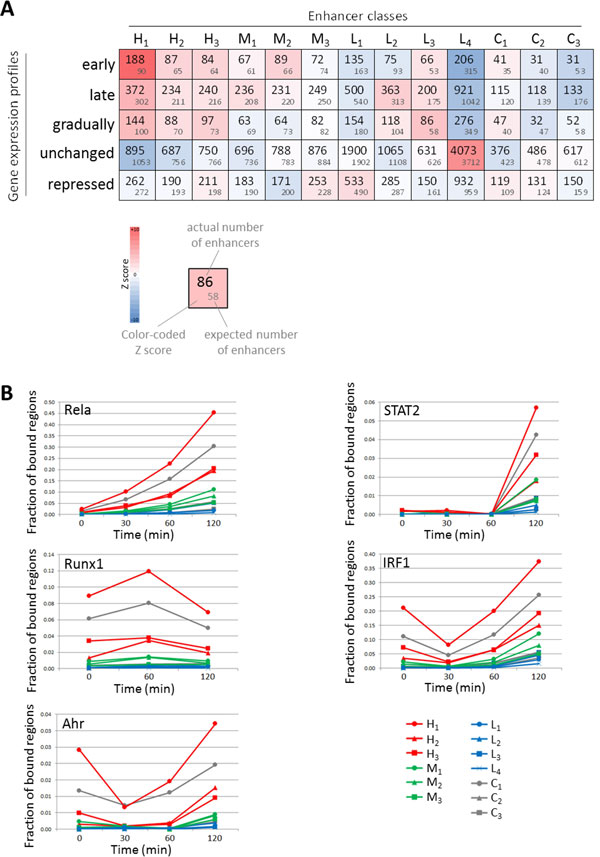
**Properties of enhancer classes**. **(A) **Figure showing the counts of enhancer regions per class (columns) associated with genes (rows) with (from top to bottom) early induction, late induction, gradual induction, no change, and repressed expression following LPS stimulation. Number in grey are expected counts based on random permutations. The colour code represents corresponding Z scores. **(B) **For Rela, STAT2, Runx1, IRF1, and Ahr, the fractions of bound enhancer regions per class are shown at different time points.

To evaluate potentially different biological functions of distinct enhancer classes, we investigated and compared a number of features of all classes. First, we evaluated correlations between the presence of enhancers of certain classes and gene expression patterns in nearby genes. We clustered genes with differential expression following LPS stimulation into 4 sets; early induction, late induction, gradual induction, and repression (Supplementary Fig. S3 in Additional file 1), and we also defined a set of 5000 genes with no change in expression as a negative control. For each set of genes we examined the class of nearby enhancers, and identified significant associations (see Materials and Methods section). We found that H_1 _enhancers were strongly associated with early induction genes (188 regions observed vs 90 expected; Z-score = 11.2) (Figure 4A), or induced genes in general (704 regions observed vs 493 expected; Z-score = 10.5). This is consistent with the observation made by Garber et al. [11]. The rest of highly bound enhancers (H_2 _and H_3_), and also a set of lowly bound enhancer (L_3_), which are defined mainly by JunB binding, showed associations with induced genes. C_1 _enhancers, on the other hand, which are bound by CTCF, had no strong association with induced genes. We observed that L_4 _enhancers were associated with genes lacking expression change (4,073 regions observed vs 3,712 expected; Z-score = 7.5), and had a corresponding tendency not to be associated with early induction genes (206 regions observed vs 315 expected; Z-score = -6.6). Second, we found differences in TF binding (as measured by ChIP-seq) induced by LPS stimulation (Figure 4B). Here too we found that enhancers of class H_1 _were preferentially bound by NF-κB subunits (NFKB1, Rel, Rela, and Relb). This was true even before stimulation but the difference became greater after stimulation: of the 4,470 H_1 _enhancer regions, 2,026 regions (45.3%) were bound by Rela 2 hours after stimulation (424 regions expected; Z-score = 88.1). A similar tendency was seen for STAT1, STAT2, and STAT3 binding. Although there was virtually no binding prior to stimulation, 255 enhancers of class H_1 _(5.7%) were bound by STAT2 (54 regions expected; Z-score = 29.3) 2 hours after stimulation. Other transcription factors also had a strong preference to bind to H_1 _enhancers, both before and after stimulation. These include Ahr, IRF1, RUNX1, Egr2, and Maff (Figure 4B and data not shown). Similar but weaker observations were made for enhancer classes H_2 _and H_3_, but also C_1 _regions. On the other hand, enhancers of classes L_1 _and L_4 _tended to lack binding by any of the investigated TFs (Figure 4B and data not shown).

Together, the above results indicate that enhancers can be separated into several classes based on their binding by a set of principal TFs in unstimulated cells, and that the classification defines to a large extent their binding by other TFs even after stimulation as well as changes in expression of nearby genes after stimulation. Our results also suggest that enhancers that are highly bound, in particular class H_1_, play a more important role in the regulation of transcription in response to immune stimulation.

### Dynamics of enhancer classes following LPS stimulation

The above results imply that the binding by a few principal TFs to enhancers prior to stimulation controls the behaviour of nearby genes after LPS stimulation. However, it is also possible that there is considerable dynamics in enhancer classes themselves after stimulation. In immune cells in particular, binding of the principal TFs to enhancers might be influenced by stimulation. To investigate the nature and extent of such changes in principal TF binding, we used the same classifiers derived prior to stimulation to classify enhancers based on their TF binding patterns at 30, 60, and 120 mins after stimulation by LPS.

**Figure 5:**
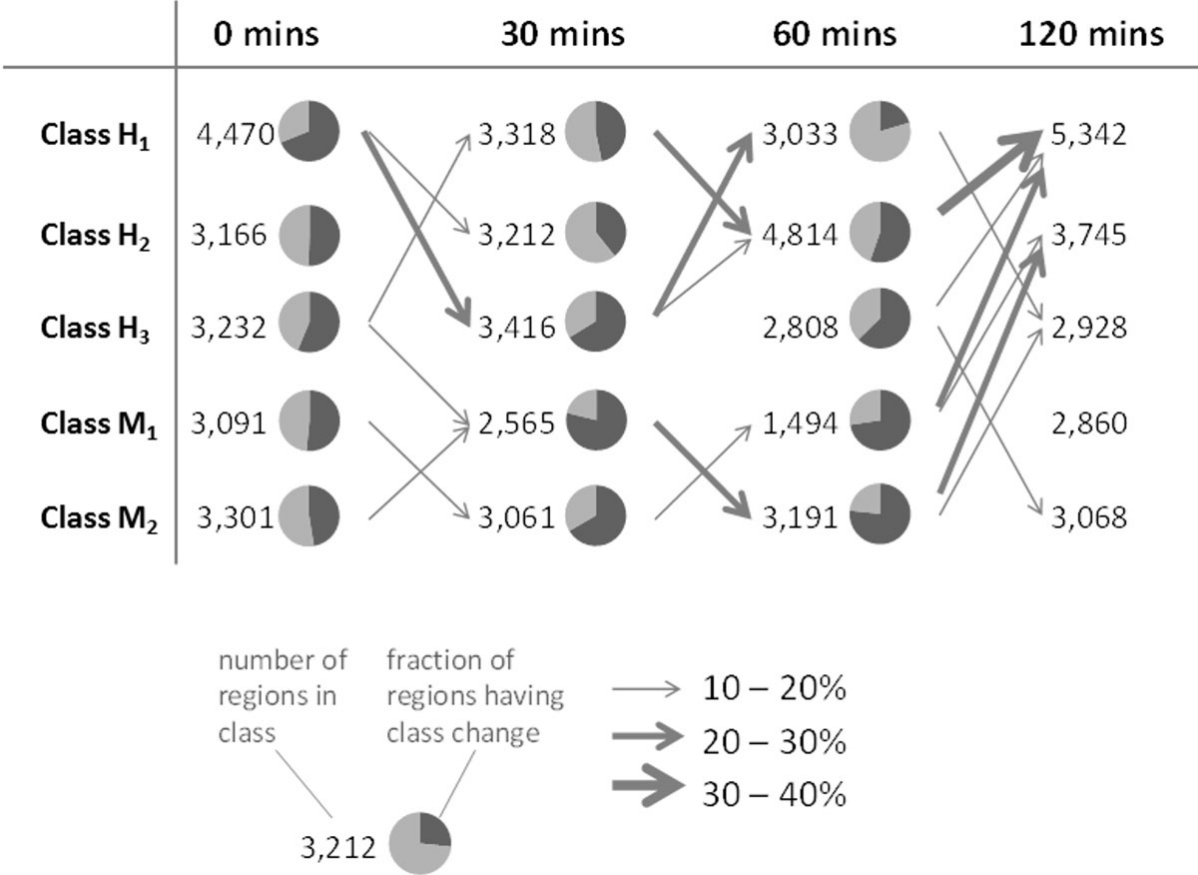
**Dynamics in enhancer classes following LPS stimulation**. For five enhancer classes (H1, H2, H3, M1, and M2) class transitions following stimulation are shown, with arrows indicating frequent transitions and the arrow thickness representing the frequency of the transition. Number indicate the number of enhancers belonging to each enhancer class at each time point, and the pie charts represent the fraction of enhancers in each class at each time point making or not making a class transition.

Our results suggest that enhancers experience extensive changes in the binding by the principal TFs following stimulation (Supplementary Fig. S4 and Supplementary Table S2 in Additional file 1). Some of the class transitions involving class H_1 _enhancers are shown in Figure 5. During the first 30 minutes following stimulation, 1,021 regions (23%) change from class H_1 _to H_3 _(losing ATF3 binding), resulting in a drop from 4,470 to 3,318 H_1 _regions. A further 904 out of 3,318 remaining H_1 _regions (27%) switch to H_2 _in the following 30 minutes (losing IRF4 binding). However, between 60 and 120 minutes following stimulation a change in the opposite direction occurs, with 1,597 H_2 _regions and 466 H_3 _regions changing to class H_1 _regions, bringing the final count to 5,342 regions. This includes 3,362 (75.2%) of the original H_1 _regions.

These results illustrate that the classification of enhancers is not static, and that enhancers are able to gain and lose binding by one or more of the principal TFs following stimulation, which, in our analysis, is reflected by class transitions.

### H_1 _enhancers that transiently lose ATF3 binding following LPS stimulation are associated with early transiently induced genes

**Figure 6:**
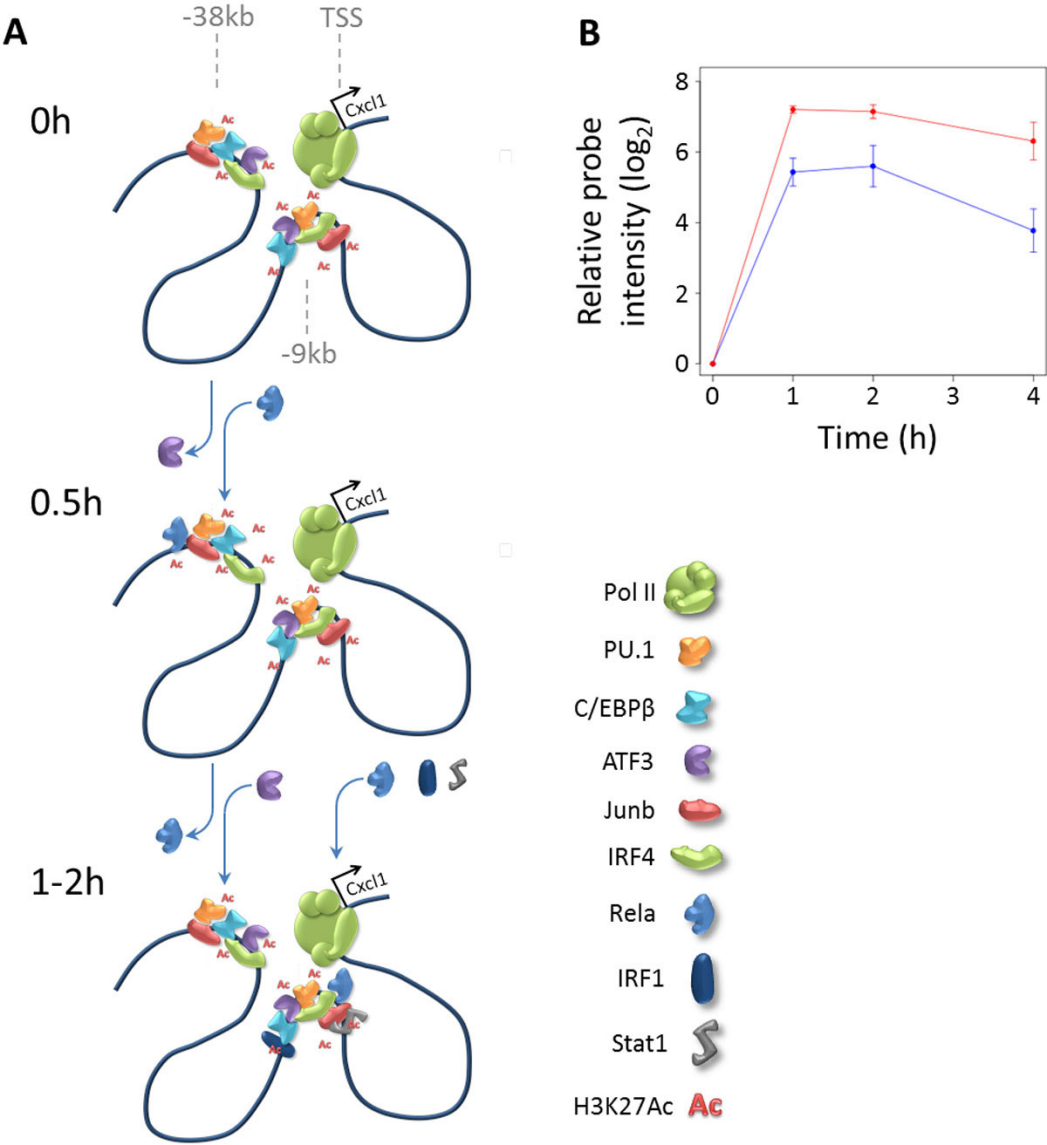
***Cxcl1 *enhancer regions illustrating role of transient loss of ATF3 binding**. **(A) **Schematic representation of the genomic region upstream of the *Cxcl1 *gene, at time points 0h, 0.5h, and 1-2h after stimulation. Two enhancers are shown, one 9kb and one 38kb upstream of the *Cxcl1 *transcription start site. The H_1 _enhancer at -38 kb loses ATF3 binding at 0.5h, and gains Rela binding. At the later time points, ATF3 binding is restored, and the enhancer at -9 kb is bound by IRF1, Rela, and STAT1. **(B) **Average relative probe intensities of the *Cxcl1 *gene are increased in ATF3 KO compared to WT cells. Average values +/- standard deviation are shown for probe 1457644_s_at (3 replicates), relative to 0h values. Similar plots are shown for two other probes in Supplementary Fig. S5A in Additional file 1.

As an illustration of the biological relevance of our enhancer classification and their changes over time, we focus here on one of the frequent changes: the transient loss of ATF3 binding at H_1 _enhancers at time point 0.5h, with a restoration of ATF3 binding at 1h, resulting in a change of H_1 _-> H_3 _-> H_1_. Of the 4,470 H_1 _enhancers, 511 follow this pattern. One example is an enhancer located about 38 kb upstream of the gene *Cxcl1*, which is illustrated in Figure 6A. This gene encodes a member of the CXC subfamily of chemokines, and plays a role in the acute inflammatory response through the recruitment of neutrophils to the site of infection [24]. Both RNA-seq data (not shown) and microarray data [25] show that *Cxcl1 *transcription is strongly induced at an early stage after LPS stimulation (Figure 6B and Supplementary Fig. S5A in Additional file 1). ATF3 has been shown to be a negative regulator in the TLR4 signalling pathway through the recruitment of histone deacetylases [25]. However, a potential regulatory role of the transient loss of ATF3 binding at enhancer regions after TLR activation in the regulation of early (and transiently) induced genes has not been described before.

The 511 enhancers transiently losing ATF3 binding are enriched around LPS-induced genes in general, but in particular in regions proximal to 141 early induced genes (28 enhancers vs 10.4 expected, Z score 5.8, Supplementary Fig. S6A in Additional file 1) and 113 transiently induced genes at time point 1h (genes with a more than 2-fold higher RPKM at 1h than at both 0h and 4h) (24 enhancers vs 7.6 expected, Z score 6.0, Supplementary Fig. S6B in Additional file 1). This enrichment is stronger than that of other subsets of H_1 _enhancers after LPS stimulation (not shown). These results suggest that the transient loss of ATF3 at these enhancers plays a role in the regulation of a subset of early and transiently induced genes. This hypothesis is supported by the enrichment of these enhancers around 111 genes that have a higher expression in a ATF3-/- KO BMDMs compared to WT BMDMs (11 enhancers vs 5.6 expected, Z score 2.2, Supplementary Fig. S6C in Additional file 1).

The above observations also fit well with the interactions between ATF3 and histone deacetylases, as suggested by Gilchrist et al. [25]. Acetylation of histones results in a more relaxed chromatin structure, increasing the accessibility of regions to TF binding, while deacetylation has the opposite effect. Higher levels of ATF3 binding might thus result in an increase of histone acetylation and in higher transcription initiation rates through the binding of additional TFs. In the case of *Cxcl1*, the loss of ATF3 at the region around -38kb coincides with binding by Rela (a component of NF- κB) and the induction of *Cxcl1 *transcription (Figure 6B and Supplementary Fig. S5A in Additional file 1). One hour after stimulation, ATF3 binding is restored, Rela binding decreases, and gene expression reaches a plateau, followed by dropping levels of mRNA. Probe intensities of *Cxcl1 *(3 probes, each with 3 replicates) after LPS stimulation are on average 2- to 4-fold higher in the ATF3 KO compared to WT cells (Figure 6B and Supplementary Fig. S5A in Additional file 1). Similar higher expression in the ATF3 KO was observed for other early-induced genes having a nearby enhancer following the H_1 _-> H_3 _-> H_1 _pattern (Supplementary Fig. S5B in Additional file 1).

### TF binding changes associated with enhancer class dynamics following stimulation

Little is known about the forces that influence TF binding at enhancers over time after stimulation of cells. Here, we examined two factors: the binding of other TFs that are only activated after stimulation, and inter-enhancer interactions.

We investigated the binding of TFs to regions where enhancer class transitions occur, and found several examples of activated TFs whose binding is correlated with enhancer class transitions. For example, enhancers of class H_1 _which change to H_3 _(thus losing binding by ATF3) tend to have lower levels of binding by Rela and IRF1 compared to regions that retain an H_1 _profile (Supplementary Fig. S7 in Additional file 1). They have lower Runx1 binding both before and after stimulation, and 120 mins. after stimulation they tend to be not bound by STAT family TFs (data not shown).

**Figure 7:**
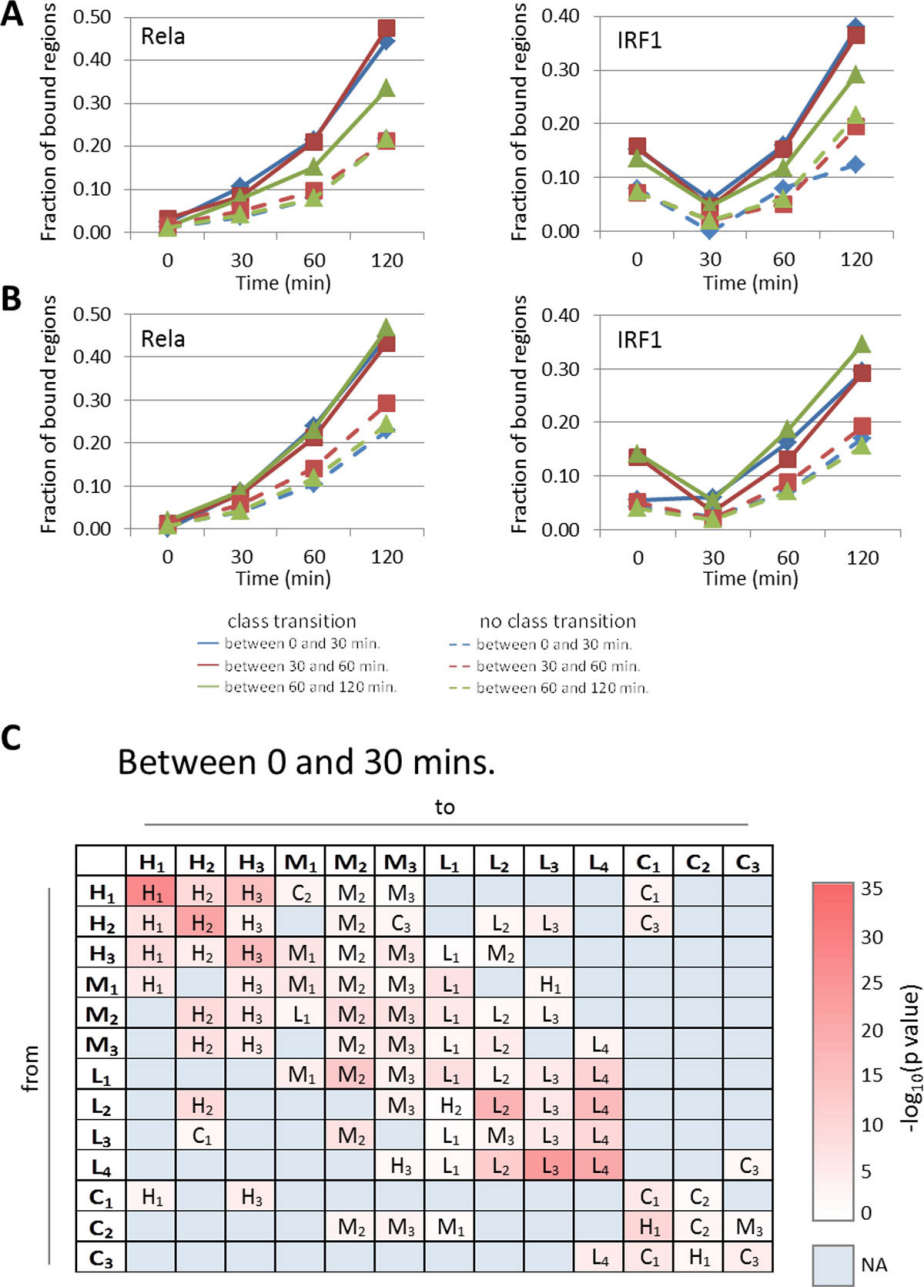
**Role of activated TFs and inter-enhancer interactions in TF binding changes after LPS stimulation**. **(A) **Two plots show the fraction of H_3 _regions bound by Rela and IRF1, respectively. Solid lines represent H_3 _regions switching to class H_1 _before 30 mins. (blue), before 60 mins. (red) and before 120 mins. (green) after stimulation. Dotted lines are for H_3 _regions not making a change between these time points. **(B) **Same as in **(A) **for H_2 _regions changing (or not changing) to class H1. **(C) **Table summarizing the positional biases between enhancers changing from one class (rows) to another (columns) between time points 0h and 0.5h. For each pair of classes, the enhancer class located most closely to the changing enhancers is shown. The colour code represents t-test p values. The same tables for time points 0.5h to 1h and 1h to 2h are shown in Supplementary Fig. S6 in Additional file 1.

On the other hand, enhancers of class H_3 _which change to class H_1 _(thus gaining binding by ATF3) between 0 and 30 mins. or between 30 and 60 mins. following stimulation show a tendency to gain binding by Rela at the time when the enhancer class transition occurs (Figure 7A). Enhancers changing from class H_3 _to H_1 _between 60 and 120 mins. on the other hand, have a tendency to be already enriched for binding by Rela compared to regions where no binding by ATF3 occurs. These regions, too, have an additional increase in Rela binding following the transition to class H1. Similarly, H_3 _regions changing to class H_1 _have a higher tendency to be bound by IRF1 compared to H_3 _regions lacking a transition to H_1 _(Figure 7A). These findings suggest a certain interaction between binding of Rela and/or IRF1 and binding of ATF3, and the formation of a H_1 _type environment.

Similar results were obtained for class H_2 _regions changing to class H_1 _(Figure 7B). In addition, enhancers of classes H_2 _and H_3 _that switch to H_1 _tend to have relatively higher binding by Maff even before stimulation and before making the class transition (data not shown), and later on these regions show relatively high binding by Rel, Relb, Runx1, E2F1, and Egr2, and after 2 hours additional binding by STAT family members. Similar changes were also observed for other time points, and on a smaller scale for regions of other classes changing to class H1.

These observations further underline the importance of the binding pattern of enhancers of class H_1_, consisting of binding by PU.1, C/EBPβ, JunB, ATF3, and IRF4, which form together an environment that can easily be bound by several other TFS, including TFs that are activated following stimulation, such as NF-κB and STAT family members. The coincidence of binding changes at certain points following stimulation also suggests a certain degree of mutual influence or cooperativity between TFs.

### Nearby enhancers influence enhancer class dynamics following stimulation

The existence of interactions between distal elements through looping is widely recognized. Several studies have described interactions, through looping, between promoters and distal sites, including enhancers [23,26]. However, interactions between pairs of enhancers, and their potential functions, are often ignored. In a final step, we evaluated whether proximally located enhancers might influence the binding of TFs after stimulation, and in particular how this might affect the formation of enhancers of class H_1_.

For each enhancer we calculated the distance to the most proximally located enhancer of each class, for each time point before and after stimulation. We picked up enhancers with a transition to a different class between time points, and compared the distances to each class of enhancers. We did the same for enhancers that did not have a transition to a different class. Finally, we compared the two sets of distances using a t-test.

In general, we found that enhancers changing to a certain class "X", tend to be located relatively proximally to an existing enhancer of class "X" (Figure 7C and Supplementary Fig. S8 in Additional file 1). For example, enhancers of class H_1 _that change to class H_3 _between time points 0 and 30 mins. tend to be more closely located to already existing enhancers of class H_3 _(median distance 49.4 kb vs 72.2 kb, t-test based p value: 3.8e-15). Similarly, between 30 and 60 mins. after stimulation, H_1 _enhancers switching to H_2 _are located more proximally to existing H_2 _enhancers (median distance 31.1 kb vs 45.2 kb, t-test based p value: 1.9e-10). Finally, between 60 and 120 mins. after stimulation, a large fraction of H_2 _enhancers change to H_1 _enhancers. These H_2 _enhancers tend to be located proximally to existing H_1 _enhancers (median distance 29.4 kb vs 50.0 kb, t-test based p value: 1.2e-35).

Based on the assumption that proximally located enhancer pairs are more likely to be interacting than distally located ones, these results suggest that the presence of enhancers belonging to one particular class make nearby enhancers more likely to change to that same class. In other words, the TFs binding to an enhancer can be influenced by TFs bound to surrounding enhancers. In the case of H_1 _enhancers, the existence of H_1 _enhancers can induce nearby enhancers to gain binding by principal TFs and to thus become H_1 _enhancers, a process that is aided by the activation and binding of specific TFs following stimulation (see previous section).

## Conclusions

In this integrative analysis we used newly obtained RNA-seq and TSS data in combination with publicly available data sets to address several questions concerning the features and dynamics of enhancers, in particular, variations in the sets of TFs binding their functional role in the regulation of transcription following stimulation, and the dynamics in binding by TFs following stimulation. For this we employed a set of datasets from myeloid APCs, allowing us to identify enhancer regions. We initially classified enhancer regions according to the TFs binding to them before stimulation, and we found a number of different enhancer classes, each defined by a different set of binding regulators. Although many regions appeared to be bound by PU.1 and C/EBPβ, several classes lacked one or even both of these regulators. Importantly, there was a strong association between genes that are induced upon LPS stimulation and a class of enhancers that are bound by PU.1, C/EBPβ, ATF3, IRF4, and JunB (H_1 _enhancers).

Interestingly, key regulators of the transcriptional response to LPS stimulation, such as NF-κB, IRFs, and STAT family TFs, bind preferentially to these H_1 _enhancer regions after stimulation. Moreover, following stimulation, there was considerable dynamics in the binding of enhancers by the principal TFs, and we observed that the acquisition of a class H_1 _enhancer binding profile tends to co-occur with the binding of NF-κB subunits, especially Rela. This suggests that the TFs bound by class H_1 _enhancers create a local environment that facilitates the binding of activated TFs, and that activated TFs contribute in the creation of this environment. Regulators that are activated at later time points, such as the STAT family TFs, also tend to favour binding to the class H_1 _enhancers.

In addition to the influence of activated TFs, our results suggest that regions with similar sets of bound TFs (such as class H_2 _enhancers, bound by PU.1, C/EBPβ, ATF3, JunB but not IRF4; and class H_3 _enhancers, bound by PU.1, C/EBPβ, IRF4, JunB but not ATF3) tend to gain IRF4 or ATF3 binding especially when they are located relatively proximally to existing class H_1 _enhancers. This too supports the existence of a local environment with increased TF binding, which might easily influence nearby enhancers, for example through looping of the DNA.

Together, our results suggest that genes can be marked for rapid induction even before stimulation by specific combinations of TFs binding to nearby enhancers, allowing for rapid initiation of transcription following stimulation. However, several levels of regulation appear to be present after stimulation, including interactions between proximally located pairs of enhancers. Such interactions might influence the induction time or stability of transcription of nearby genes, which are important factors in the response against pathogens. Future analyses using Carbon-Copy Chromosome Conformation Capture (5C) or related techniques will be necessary to further investigate the interactions between enhancers with particular TF binding profiles and their changes over time.

Our results confirmed that a large fraction of regions with enhancer-like features in myeloid APCs are bound by PU.1 and C/EBPβ, as has recently been reported [8,9,11]. However, we showed that the specific properties of the enhancers are defined by the specific subset of TFs binding to them, even before stimulation. Our integrative study underscores the importance of detailed analysis of high-throughput sequencing data and how it can reveal findings that are obscured when averaging over all enhancers.

## List of abbreviations used

APC antigen presenting cell

BMDC bone marrow-derived dendritic cell

BMDM bone marrow-derived macrophage

DC dendritic cell

DDBJ DNA Data Bank of Japan

KO knock-out

LPS lipopolysaccharide

Pol2 RNA polymerase II

RPKM reads per kilobase per million reads

TF transcription factor

TSS transcription start site

WT wild-type

## Competing interests

The authors declare that they have no competing interests.

## Authors' contributions

AV carried out data processing and bioinformatics analysis. ST and DMS participated in the bioinformatics analysis and helped to draft the manuscript. OT and YS prepared biological samples and sequencing data, and assisted in the analysis and interpretation of data. All authors read and approved the final manuscript.

## Supplementary Material

Additional file 1 **Supplementary material - (PDF file)**. File containing description of supporting analyses as well as supplementary tables and figures. Click here for file

## Declarations

Publication costs for this article were funded by the corresponding authors' institution.

This article has been published as part of *BMC Genomics *Volume 15 Supplement 10, 2014: Proceedings of the 25th International Conference on Genome Informatics (GIW/ISCB-Asia): Genomics. The full contents of the supplement are available online at http://www.biomedcentral.com/bmcgenomics/supplements/15/S10.
