# Supplementary material for: Dynamics of enhancers in myeloid antigen presenting cells upon LPS stimulation
Source: BMC Genomics. 2014 Dec 12;15(Suppl 10):S4. doi: 10.1186/1471-2164-15-S10-S4 (PMC4304210; doi:10.1186/1471-2164-15-S10-S4)
Supplement: Additional file 1 — Supplementary material - (PDF file). File containing description of supporting analyses as well as supplementary tables and figures. [file 1471-2164-15-S10-S4-S1.pdf]

Supplementary Material for Dynamics of enhancers in myeloid antigen presenting cells upon LPS stimulation

Supporting analysis of "active promoter" and "enhancer" region properties

We compared a number of properties of "promoter" and "enhancer" regions as defined in the main paper. We observed that both clusters contained high levels of PU.1 and C/EBPβ ChIP-seq tags, but Pol2 binding was restricted mainly to the "promoter" cluster (Supplementary Fig. 1B). CpG scores and GC content (not shown) were markedly higher in "promoter" regions (and "repressed 1" regions). The distribution of distances to the most proximal RefSeq TSS showed that "promoter" regions tended to be located proximally to known TSSs (89.8% were located < 5kb from a known Refseq TSS), while "enhancer" regions were typically located more than several kbs away from known TSSs (87.2% were located > 5kb from a known Refseq TSS). Using Refseq gene annotations, we found that 23.3% of "promoter" regions were located around known TSS regions or within 5'UTRs of known genes in contrast to only 0.7% members of the "enhancer" cluster (Supplementary Fig. 1C). The vast majority (94.4%) of "enhancer" members, on the other hand, were located in intergenic regions in contrast to 57.3% for "promoters". Together, these features support the nomenclature of these regions.

Analysis of TFBS motif enrichment in enhancer classes

Genomic regions corresponding to all enhancer regions were extracted from the mouse genome (version mm9). The central 5 bins (corresponding to the region -500 to +500 surrounding the region center) of all enhancers was scanned, using a collection of 543 position weight matrices (PWMs) collected from the Jaspar database (Mathelier *et al.*, 2014). For each PWM a threshold score was set in such a way that it results in about 1 predicted site per 5kb in the genome.

For each PWM, motif enrichment in each of the enhancer classes was evaluated by comparing the number of regions having 1 or more predicted TFBSs, with a large set of randomly selected genomic sequences with similar GC content. Briefly, for each enhancer region, for each of the 5 central bins the GC content was calculated and a randomly selected region of similar GC content (+/- 5%) was picked. This was repeated 10 times, thus constructing a large control set with similar GC content as the input enhancer sequences. Enrichment of TFBSs of a PWM was then evaluated by comparing the number of enhancer regions containing 1 or more predicted TFBS motifs, with the ratio of randomly selected genomic regions of similar GC content containing 1 or more predicted TFBS motifs, using a binomial distribution. This way, biases in motif enrichment caused by GC content were avoided.

The 20 motifs with the most significant enrichment in 1 or more enhancer classes were selected, and clustered according to similarity in enrichment pattern, based on the Euclidean distance in fold enrichment. This clustering was visualized in a heatmap (Supplementary Fig. S2). The colors of the heatmap represent the fold enrichment of each of these TFBS motifs in each enhancer class.

The results of the TFBS motif analysis fit well with the ChIP-seq data used to classify the enhancers (see Fig. 3 in the main manuscript). The PU.1 (Sfpi1) binding motif is enriched in all enhancer classes, except L_2, L_3, L_4, and C_3. Other ETS family motifs show a similar pattern. The CEBPA, which is similar to the motif recognized by C/EBPβ, is enriched in the classes that have C/EBPβ binding according to the ChIP-seq data. The enrichment of the motif is especially strong in the class L_2 enhancers, which are only bound by C/EBPβ and not by other "principal" TFs. In a similar way, AP-1 motifs (AP-1, Jundm2 (2), and Fos) are especially enriched in class L_3 enhancers, and CTCF motifs have especially high enrichment in C_3 enhancers.

Supplementary Table S1: TF binding to enhancer regions and active promoters in resting BMDCs (before LPS stimulation). For each TF the percentage of all enhancers and all promoters it binds to is shown.

	Bound enhancers (%)
PU1	38.0
Cebpb	26.0
Junb	14.1
Irf4	12.1
Ctcf	8.9
Atf3	8.4
Irf1	2.9
Maff	2.7
Egr2	1.3
Runx1	1.3
Irf2	0.7
Rela	0.4
Ahr	0.3
E2f4	0.1
Nfkb1	0.1
E2f1	0.1
Hif1a	0.1
Ets2	0.0
Egr1	0.0
Stat2	0.0
Rel	0.0
Relb	0.0
Stat1	0.0
Stat3	0.0

Supplementary Table S2: Table showing the mean percentage of regions within each enhancer class that makes a transition to a different class between the 4 time points, following stimulation.

Enhancer class index	Average percentage class transitions
H_1	38.2
H_2	48.4
H_3	61.7
M_1	67.7
M_2	70.7
M_3	57.0
L_1	37.2
L_2	42.3
L_3	51.4
L_4	15.2

**C_1**	31.9
**C_2**	36.0
**C_3**	18.9

Supplementary Figure S1: Properties of clusters of genomic regions. **(A)** Graphs representing the mean value of the features used for clustering (H3K27me3, H3K4me1, H3K4me3, and TSS-seq tags) over the region -2kb to +2kb around the center of regions for the 4 classes. Red: class 1 (active promoters), blue: class 2 (enhancers), green: class 3 (repressed 1), and yellow: class 4 (repressed 4). **(B)** Graphs representing additional features over the region -2kb to +2kb around the center of regions for the 4 classes. Red: class 1, blue: class 2, green: class 3, and yellow: class 4. **(C)** Pie charts representing the association of class 1 and class 2 regions with genomic features.

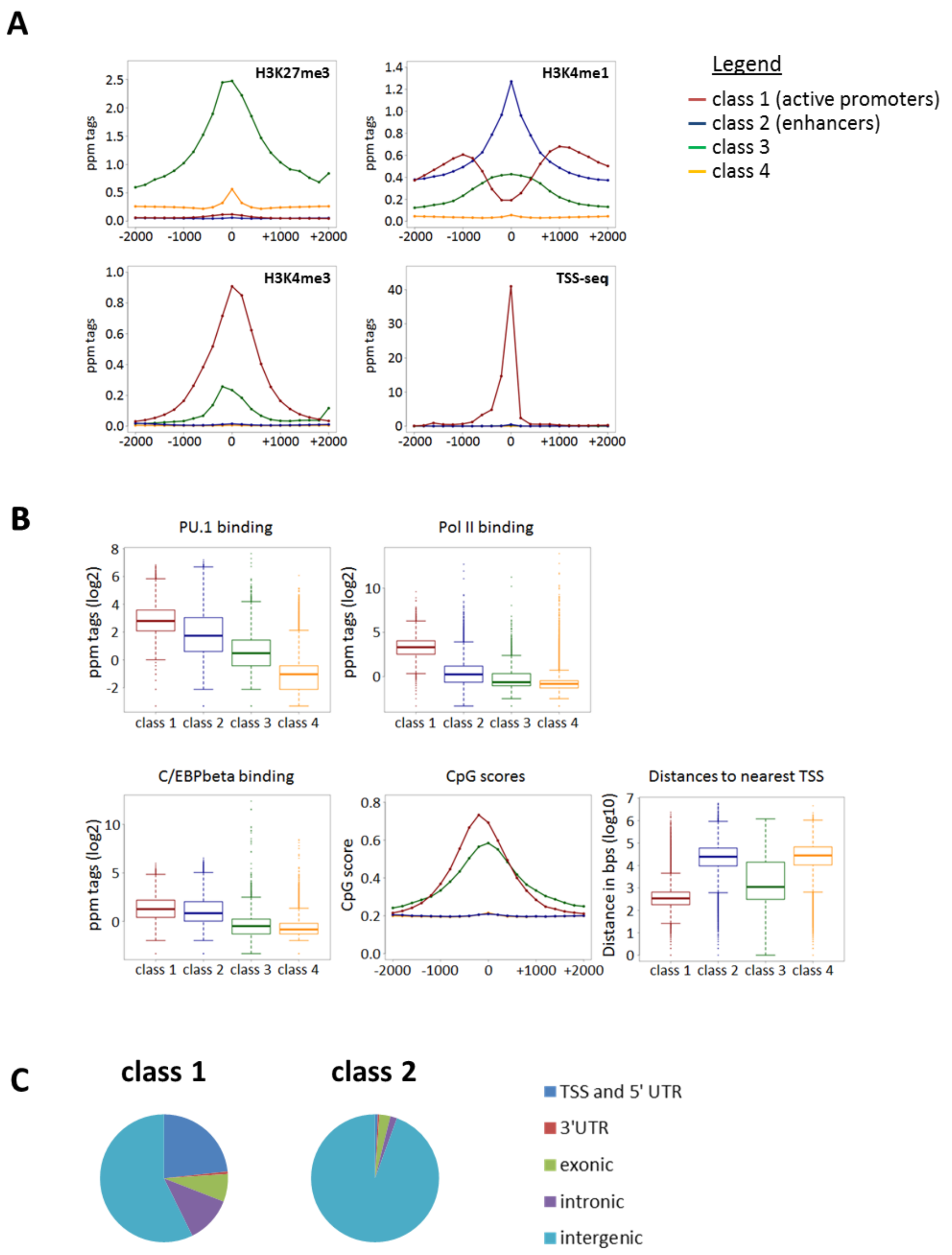

Supplementary Figure S2: Heatmap representing the TFBS motif enrichment of the 20 TFBS motifs (rows) with the most significant enrichment in 1 or more enhancer classes (columns). Motifs were clustered according to the similarity in their enrichment pattern. Colors represent the fold enrichment of each of the motifs in each enhancer class. Enhancer classes are indicated below the heatmap. Jaspar TF IDs are shown at the right.

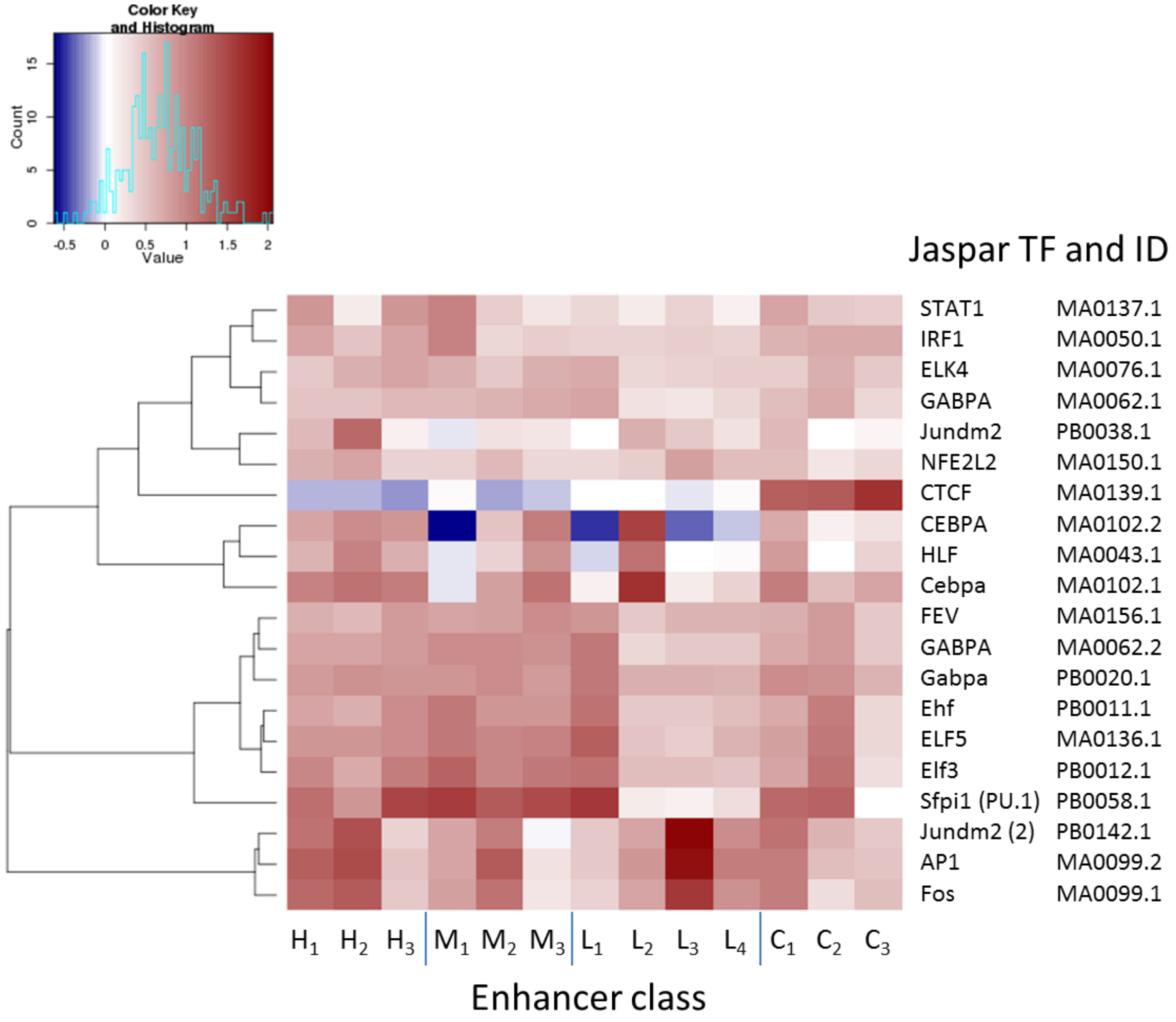

Supplementary Figure S3: Heatmap of genes with differential expression following LPS stimulation (RNA-seq data). Genes are clustered into 4 classes using hierarchical clustering (see Methods). For each cluster a short description is shown, as well as the number of genes it contains.

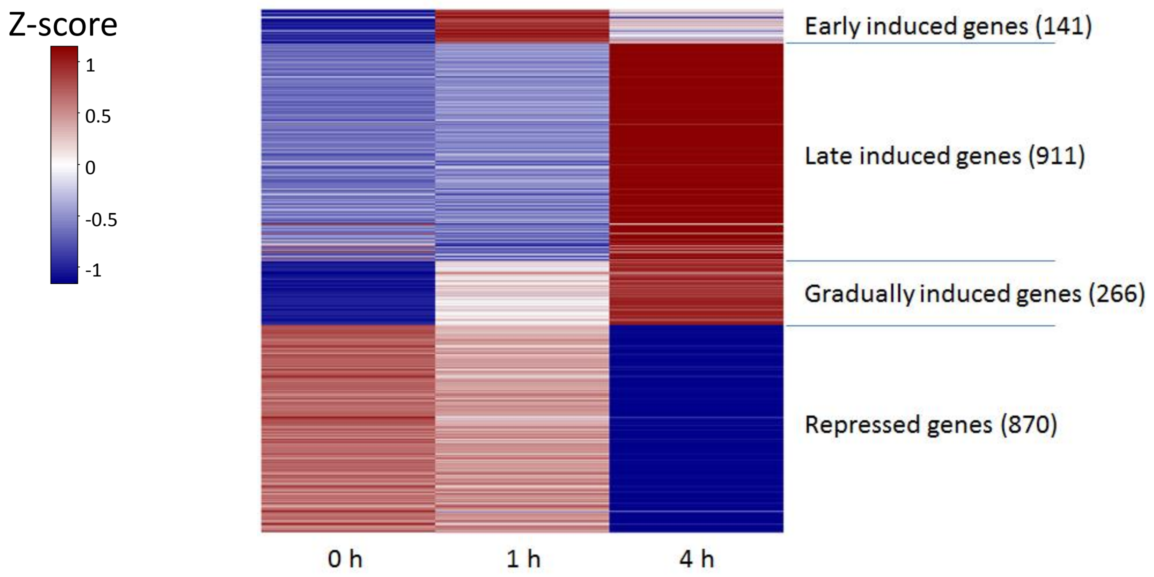

Supplementary Figure S4: Overview of class transitions between enhancer classes between time points after LPS stimulation. For the period 0 to 30 mins., 30 to 60 mins., 60 to 120 mins., the fraction of each enhancer class (rows) making a transition to another enhancer class (columns) is shown. For easier interpretation values are colour coded (white: low, red: high). The average over the 3 ransitions is shown in the bottom table

0 to 30 min.

from \ to	H_1	H_2	H_3	M_1	M_2	M_3	L_1	L_2	L_3	L_4	C_1	C_2	C_3
H_1		0.10	0.23	0.07	0.02	0.03	0.00	0.01	0.01	0.00	0.01	0.00	0.00
H_2	0.06		0.05	0.01	0.09	0.12	0.01	0.09	0.05	0.00	0.02	0.00	0.00
H_3	0.15	0.04		0.12	0.04	0.15	0.02	0.02	0.01	0.00	0.01	0.00	0.00
M_1	0.03	0.01	0.06		0.11	0.03	0.22	0.00	0.02	0.01	0.01	0.01	0.00
M_2	0.01	0.12	0.08	0.03		0.21	0.13	0.04	0.05	0.01	0.00	0.01	0.00
M_3	0.01	0.05	0.05	0.01	0.05		0.17	0.12	0.01	0.03	0.00	0.01	0.00
L_1	0.00	0.00	0.00	0.01	0.09	0.04		0.01	0.02	0.17	0.00	0.01	0.00
L_2	0.00	0.05	0.01	0.00	0.01	0.05	0.02		0.07	0.18	0.00	0.00	0.01
L_3	0.01	0.04	0.01	0.01	0.07	0.01	0.06	0.08		0.21	0.00	0.00	0.01
L_4	0.00	0.00	0.00	0.00	0.00	0.00	0.05	0.05	0.03		0.00	0.00	0.01
C_1	0.06	0.03	0.04	0.02	0.01	0.02	0.00	0.01	0.00	0.00		0.07	0.02
C_2	0.00	0.00	0.00	0.02	0.03	0.03	0.04	0.00	0.00	0.00	0.16		0.12
C_3	0.00	0.00	0.00	0.00	0.00	0.00	0.01	0.02	0.01	0.07	0.04	0.06	

30 to 60 min.

from \ to	H_1	H_2	H_3	M_1	M_2	M_3	L_1	L_2	L_3	L_4	C_1	C_2	C_3
H_1		0.27	0.08	0.04	0.01	0.02	0.00	0.00	0.00	0.00	0.03	0.00	0.00
H_2	0.07		0.09	0.03	0.03	0.06	0.00	0.05	0.03	0.00	0.02	0.00	0.00
H_3	0.22	0.16		0.02	0.09	0.11	0.02	0.02	0.01	0.00	0.01	0.01	0.00
M_1	0.05	0.04	0.07		0.27	0.05	0.25	0.00	0.02	0.00	0.01	0.03	0.00
M_2	0.01	0.09	0.05	0.13		0.08	0.21	0.01	0.05	0.01	0.00	0.02	0.00
M_3	0.01	0.10	0.12	0.01	0.07		0.14	0.08	0.01	0.01	0.01	0.01	0.00
L_1	0.00	0.00	0.00	0.02	0.06	0.05		0.02	0.02	0.15	0.00	0.01	0.00
L_2	0.00	0.06	0.02	0.00	0.01	0.05	0.03		0.07	0.21	0.00	0.00	0.01
L_3	0.00	0.07	0.01	0.02	0.05	0.01	0.06	0.08		0.16	0.00	0.00	0.01
L_4	0.00	0.00	0.00	0.00	0.00	0.00	0.06	0.04	0.03		0.00	0.00	0.01
C_1	0.03	0.03	0.03	0.01	0.01	0.01	0.00	0.00	0.00	0.00		0.22	0.04
C_2	0.00	0.00	0.00	0.01	0.01	0.02	0.05	0.00	0.00	0.01	0.07		0.12
C_3	0.00	0.00	0.00	0.00	0.00	0.00	0.01	0.01	0.01	0.05	0.03	0.07	

60 to 120 min.

from \ to	H_1	H_2	H_3	M_1	M_2	M_3	L_1	L_2	L_3	L_4	C_1	C_2	C_3
H_1		0.03	0.13	0.02	0.01	0.00	0.00	0.00	0.00	0.00	0.02	0.00	0.00
H_2	0.33		0.06	0.01	0.07	0.03	0.01	0.02	0.01	0.00	0.01	0.00	0.00
H_3	0.17	0.08		0.04	0.20	0.09	0.01	0.03	0.00	0.00	0.01	0.00	0.00
M_1	0.26	0.17	0.06		0.08	0.02	0.10	0.00	0.02	0.00	0.01	0.00	0.00
M_2	0.03	0.04	0.11	0.24		0.06	0.19	0.01	0.05	0.01	0.00	0.01	0.00
M_3	0.03	0.06	0.12	0.04	0.16		0.10	0.08	0.01	0.01	0.00	0.01	0.00
L_1	0.00	0.00	0.01	0.12	0.03	0.07		0.02	0.03	0.13	0.00	0.01	0.00
L_2	0.01	0.06	0.02	0.00	0.04	0.06	0.02		0.04	0.14	0.00	0.00	0.01
L_3	0.02	0.10	0.02	0.03	0.05	0.01	0.05	0.13		0.13	0.00	0.00	0.01
L_4	0.00	0.00	0.00	0.00	0.00	0.01	0.06	0.06	0.03		0.00	0.00	0.01
C_1	0.08	0.06	0.02	0.00	0.01	0.00	0.00	0.01	0.00	0.00		0.07	0.04
C_2	0.00	0.00	0.02	0.03	0.02	0.02	0.03	0.00	0.01	0.00	0.13		0.10
C_3	0.00	0.00	0.00	0.00	0.00	0.00	0.01	0.01	0.01	0.04	0.02	0.07	

average

from \ to	H_1	H_2	H_3	M_1	M_2	M_3	L_1	L_2	L_3	L_4	C_1	C_2	C_3
H_1		0.13	0.15	0.04	0.01	0.02	0.00	0.00	0.00	0.00	0.02	0.00	0.00
H_2	0.16		0.07	0.02	0.06	0.07	0.01	0.05	0.03	0.00	0.02	0.00	0.00
H_3	0.18	0.09		0.06	0.11	0.12	0.02	0.02	0.01	0.00	0.01	0.00	0.00
M_1	0.11	0.08	0.06		0.15	0.03	0.19	0.00	0.02	0.01	0.01	0.01	0.00
M_2	0.02	0.08	0.08	0.13		0.12	0.17	0.02	0.05	0.01	0.00	0.01	0.00
M_3	0.02	0.07	0.10	0.02	0.09		0.13	0.09	0.01	0.02	0.00	0.01	0.00
L_1	0.00	0.00	0.01	0.05	0.06	0.05		0.02	0.02	0.15	0.00	0.01	0.00
L_2	0.00	0.06	0.02	0.00	0.02	0.06	0.02		0.06	0.18	0.00	0.00	0.01
L_3	0.01	0.07	0.01	0.02	0.06	0.01	0.06	0.10		0.17	0.00	0.00	0.01
L_4	0.00	0.00	0.00	0.00	0.00	0.00	0.06	0.05	0.03		0.00	0.00	0.01
C_1	0.06	0.04	0.03	0.01	0.01	0.01	0.00	0.01	0.00	0.00		0.12	0.03
C_2	0.00	0.00	0.01	0.02	0.02	0.02	0.04	0.00	0.01	0.00	0.12		0.11
C_3	0.00	0.00	0.00	0.00	0.00	0.00	0.01	0.01	0.01	0.05	0.03	0.07	

.

Supplementary Figure S5: (A) Average probe intensities of the Cxcl1 gene are increased in ATF3 KO compared to WT cells. Average values +/- standard deviation are shown for probes 1441855_x_at and 1419209_at (3 replicates each) relative to 0h values. The same plot for probe 1457644_s_at is shown in Fig. 6B of the main manuscript. **(B)** Averaged differences between ATF3 KO and WT are summarized for the set of early induced genes that are located proximally to enhancers following the H_1 -> H_3 -> H_1 pattern. Early induced genes were defined as genes with at least a 2 fold induction at time points 1h or 2h after LPS stimulation compared to before stimulation (Ghilchrist *et al.* data). Values are $\log_2$(WT/KO) with color codes reflecting the value of the difference. Values with absolute values ≥ 0.5 are marked in green. While many genes have higher expression in the KO (especially at 1-2 h), only few have higher expression in the WT.

A

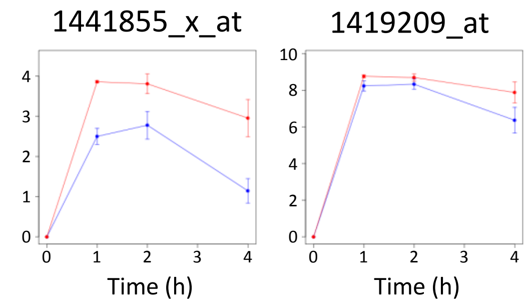

B

Time (h)

	0	1	2	4
**Cxcl3**	0.03	-2.15	-1.68	-1.22
**Cited2**	-1.36	-1.25	-0.93	-0.95
**Tlr2**	-0.78	-1.39	-0.93	-0.38
**Jdp2**	-0.85	-1.37	-0.60	0.91
**Ets2**	-0.48	-1.02	-1.21	-0.75
**Hivep2**	-0.94	-0.12	-1.59	0.11
**Cxcl1**	0.00	-1.25	-1.01	-1.98
**Map3k8**	-0.35	-0.21	-1.62	0.10
**Cdk6**	-0.60	-0.88	-0.52	0.24
**Rapgef2**	-0.54	-0.71	-0.41	-0.28
**Ptger4**	-0.53	-0.32	-0.57	0.20
**Rnf19b**	-0.27	-0.66	-0.44	0.00
**Plk2**	-0.45	-0.40	-0.48	-0.97
**Ier5**	-0.47	-0.60	-0.14	-0.31
**Pdpn**	-0.21	-0.57	-0.36	0.78
**Baz1a**	-0.22	-0.74	-0.11	0.03
**Lcp2**	0.00	-0.30	-0.71	-0.08
**Tiparp**	0.01	-0.31	-0.54	0.03
**Kpna3**	0.04	0.00	-0.65	-0.21
**Bcl2l11**	0.14	-0.48	-0.14	0.29
**Prdm1**	0.40	-0.27	-0.57	0.68
**Jhdm1d**	-0.32	0.33	-0.26	-0.05
**St3gal1**	0.56	-0.04	-0.71	0.10
**Coq10b**	0.07	-0.16	0.02	0.18
**Sgms1**	0.42	-0.34	-0.05	0.38
**Ier3**	0.09	-0.53	0.47	-0.05
**Adora2b**	0.07	-0.05	0.34	2.10
**Il10**	0.19	0.09	0.21	0.11
**Btg1**	0.39	0.40	0.15	0.14
**Etv3**	0.30	0.66	0.40	0.23
**Fos**	0.84	0.27	0.28	0.96

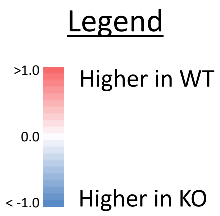

Supplementary Figure S6: Role of H_1 enhancers that transiently lose ATF3 binding 30 mins. after LPS stimulation. Three histograms are shown with the actual count of enhancers (red), as well as the expected counts based on randomizations (grey bars). Actual and expected counts of enhancers are shown proximal to 135 early induced genes (A), proximal to 113 transiently induced genes (B), and proximal to 111 genes with higher expression in ATF3 KO compared to WT (C).

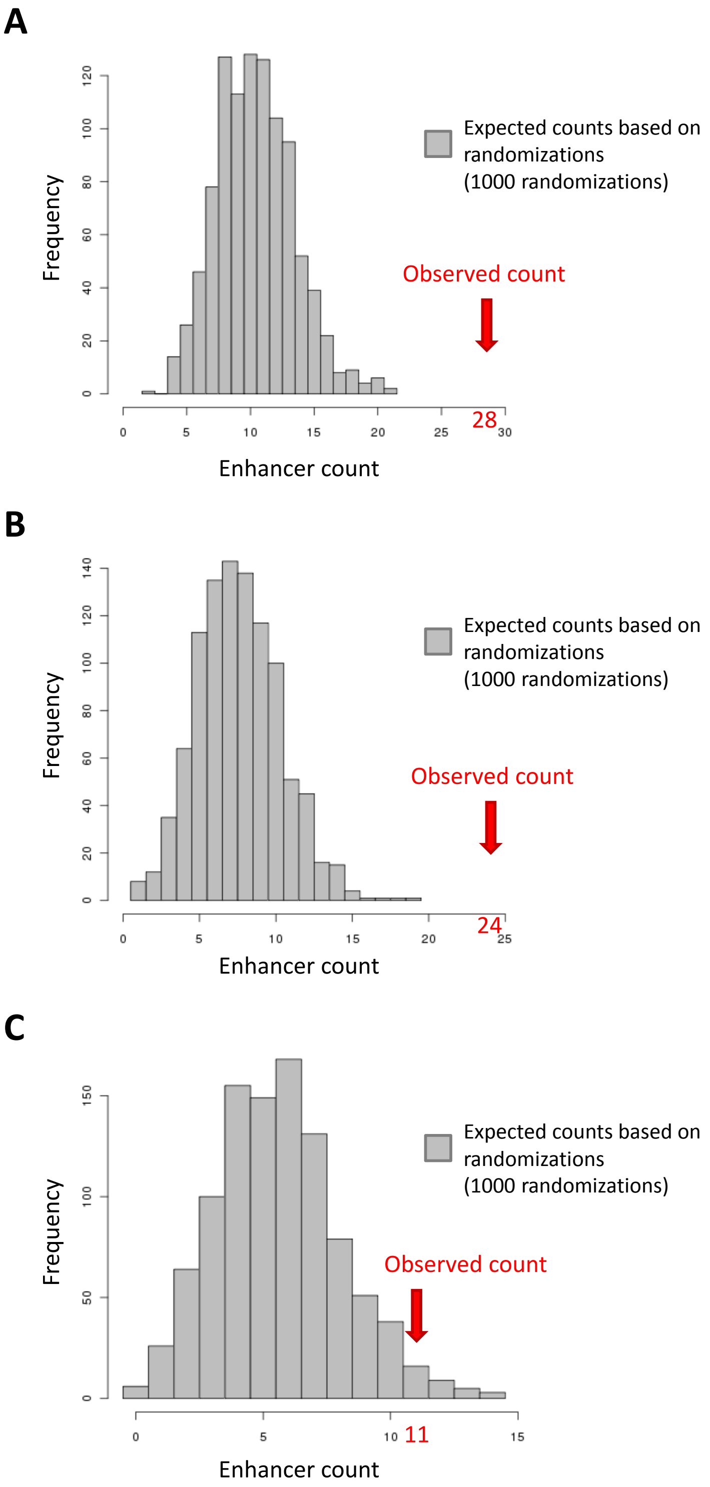

Supplementary Figure S7: Role of activated TFs and inter-enhancer interactions in TF binding changes after LPS stimulation. (A) Two plots show the fraction of H_1 regions bound by Rela and IRF1, respectively. Full lines represent H_1 regions switching to class H_3 before 30 mins. (blue), before 60 mins. (red) and before 120 mins. (green) following stimulation. Dotted lines are for H_1 regions not making a change between these time points. (B) Same as in (A) for H_1 regions changing (or not changing) to class H_2. In general the H_1 regions not switching to H_2 or H_3 tend to be more bound by Rela or IRF1. See also Fig. 7A and B in the main text.

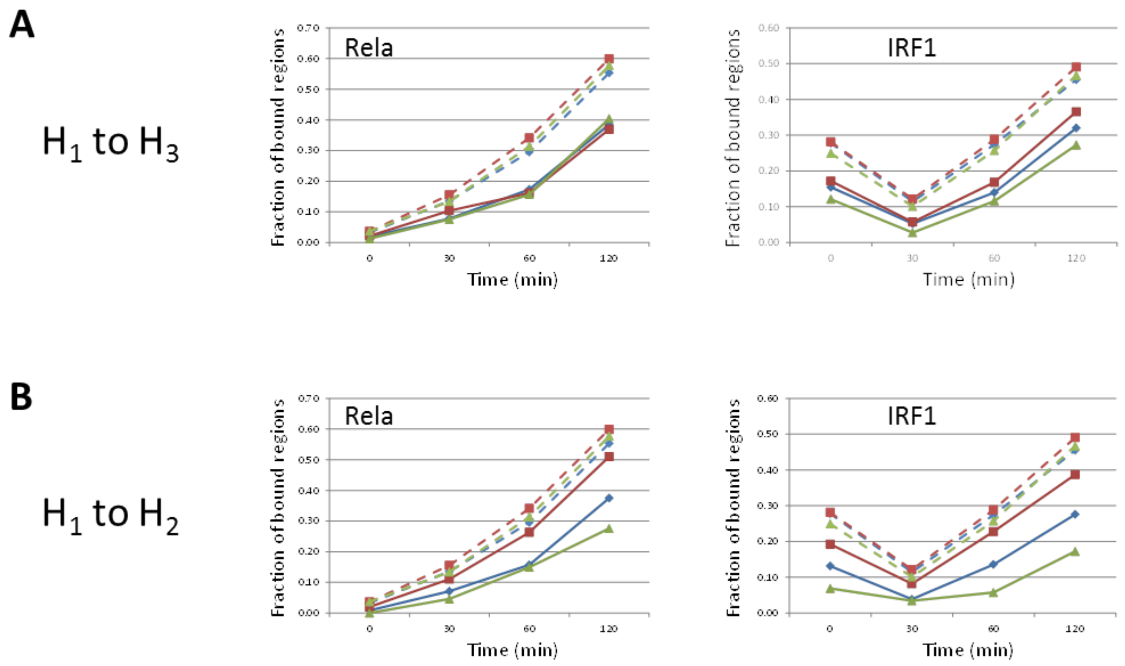

Supplementary Figure S8: Table summarizing the positional biases between enhancers changing from one class (rows) to another (columns) between time points 30 and 60 mins. and between 60 and 120 mins. For each pair of classes, the enhancer class located most proximally to the changing enhancers is shown. The colour code represents t-test p values. See also Fig. 7C in the main text.

Between 30 and 60 mins.

from \ to	H_1	H_2	H_3	M_1	M_2	M_3	L_1	L_2	L_3	L_4	C_1	C_2	C_3
H_1	H_1	H_2	L_4	M_1		M_3					C_1		
H_2	H_1	H_2	H_3	C_1	M_2	M_3		L_2	L_3		C_2		
H_3	H_1	H_2	H_3	M_1	M_2	M_3	L_4	L_2					
M_1	H_1	H_2	H_3	M_1	M_2	M_3	L_1		L_3			M_3	
M_2		H_2	H_3	M_1	M_2	M_3	L_1		L_3			C_2	
M_3		H_2	H_3		M_2	M_3	L_1	L_2	L_4	L_4		C_2	
L_1				M_1	M_2	M_3	L_1	L_2	L_3	L_4		C_2	
L_2		H_2	H_3			M_3	L_1	L_2	L_3	L_4			
L_3		H_2		M_1	M_2		L_1	L_2	L_3	L_4			
L_4							L_1	L_2	L_3	L_4			C_3
C_1	L_1	L_3	H_1								C_1	C_2	L_2
C_2							L_2				C_1	C_2	M_3
C_3										L_1	H_3	H_3	C_3

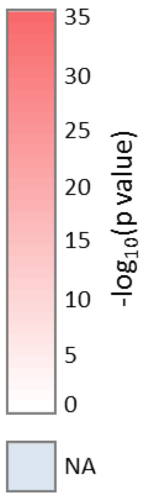

Between 60 and 120 mins.

from \ to	H_1	H_2	H_3	M_1	M_2	M_3	L_1	L_2	L_3	L_4	C_1	C_2	C_3
H_1	H_1	H_2	H_3								M_2		
H_2	H_1	H_2	H_3		M_2	C_3		L_2	L_4				
H_3	H_1	H_2	H_3	M_1	M_2	M_3		L_2					
M_1	H_1	H_2	H_3	M_1	M_2		C_3						
M_2	H_1	H_2	H_3	M_1	M_2	M_3	L_1		L_3				
M_3	H_1	H_2	H_3	M_1	M_2	M_3	L_1	L_2					
L_1			H_3	M_1	H_3	M_3	L_1	L_2	L_3	L_4		C_2	
L_2		H_2	H_3		M_2	M_3	L_1	L_2	L_3	L_4			
L_3	H_1	H_2	H_3	M_1	L_2		L_1	L_2	L_3	L_4			
L_4						H_1	L_1	L_2	L_3	L_4			L_3
C_1	H_1	L_1									C_1	C_2	L_4
C_2				M_1			L_4				H_1	C_2	C_3
C_3										L_4	C_1	C_2	C_3
